# Long-term outcomes and risk factors of thyroid dysfunction during pegylated interferon and ribavirin treatment in patients with chronic hepatitis C infection in Taiwan

**DOI:** 10.1186/s12902-019-0362-7

**Published:** 2019-04-05

**Authors:** Yu-Kang Chang, Yuan-Tsung Tseng, Kou-Huang Chen, Kow-Tong Chen

**Affiliations:** 1 Department of Radiology, Chi Mei Medical Center, Liouying, Tainan, Taiwan; 2 Department of Medical Research, Tainan Municipal Hospital (managed by Show Chwan Medical Care Corporation), Tainan, Taiwan; 3 School of Mechanical & Electrical Engineering, Sanming University, Sanming, Fujian Province China; 4 Department of Occupational Medicine, Tainan Municipal Hospital (managed by Show Chwan Medical Care Corporation), Tainan, Taiwan. No. 670, Chongde Road, East District, Tainan, Taiwan; 5 Department of Public Health, College of Medicine, National Cheng Kung University, Tainan, Taiwan

**Keywords:** Hepatitis C infection, Thyroid dysfunction, Epidemiology, Interferon, Ribavirin, Morbidity

## Abstract

**Background:**

This study aimed to investigate the occurrence and risk factors of thyroid dysfunction (TD) in patients with chronic hepatitis C (CHC) infection in Taiwan.

**Methods:**

The data in this study were obtained from the Taiwan National Health Insurance Research (Taiwan NHIR) database between 2001 and 2013. CHC patients treated with pegylated interferon/ribavirin (PEG-IFN/RBV) were enrolled as case patients, and nontreated CHC patients were enrolled as controls and were matched at a control:case ratio of 3:1 by index date, age (± 3 years), and sex. We compared the cumulative incidence of TD between the cohorts at follow-up until 2013.

**Results:**

During the study period, 3810 cases and 9393 controls were included in the study. Among the study subjects, 173 (4.5%) case patients and 244 (2.6%) controls were diagnosed with TD during the follow-up period. The types of TD were hypothyroidism (42.9%), hyperthyroidism (31.2%), and thyroiditis (25.9%). Compared to controls during the 13-year follow-up, patients treated with PEG-IFN/RBV had a higher incidence rate of TD (*P* < 0.0001), as determined using the Kaplan-Meier method. Cox proportional hazards regression analysis showed that female sex (adjusted hazard ratio (HR): 1.49; 95% confidence interval (CI): 1.23–1.75; *P* < 0.001), treatment with PEG-IFN/RBV (HR: 1.68; 95% CI: 1.38–2.06; *P* < 0.001), hyperlipidemia (HR: 1.38; 95% CI: 1.12–1.71; *P* < 0.001), and past history of goiter (HR: 6.40; 95% CI: 5.00–8.18; *P* < 0.001) were independent predictors for the development of TD.

**Conclusions:**

PEG-IFN/RBV treatment may be an independent risk factor for thyroid dysfunction among patients with hepatitis C virus (HCV) infection. Monitoring thyroid function keenly during PEG-IFN/RBV therapy in patients with chronic HCV infection is recommended for clinicians, especially for female patients and for patients with a history of hyperlipidemia and goiter.

## Background

Approximately 3% of the world’s population suffers from hepatitis C virus (HCV) infection, and approximately 70% of HCV patients will develop chronic hepatitis C (CHC) [1]. HCV is both a hepatotropic and lymphotropic virus. Both the liver and various nonhepatic tissues and organs may be affected during infection with HCV [1, 2]. HCV infection may cause complex diseases with many etiologically unrelated symptoms; therefore, the concept of systemic HCV infection has emerged [1–3]. Approximately 20–35% of chronically infected patients progress to cirrhosis and have a higher risk of developing hepatocellular carcinoma [4, 5]. Immunological reactions as well as virus invasion and replication in the affected extrahepatic tissues and organs are the main extrahepatic manifestations of HCV infection [6]. Previous studies have demonstrated that there is a high risk for thyroid autoimmunity and thyroid dysfunction (TD) in patients with HCV infection [7, 8].

Both singly and in combination with other drugs, interferon-based drugs have been commonly used to treat patients with HCV infection [9, 10]. Ribavirin (RBV) is a common drug and is often used with interferon (IFN) to treat patients with HCV infection [11]. RBV is an analog of guanosine, which induces the production of Th1 cytokines in the immune response against HCV infection [12]. The combined use of IFN and RBV stimulates the immune system response and eradicates HCV from the body [13]. However, this treatment has several side effects, such as influenza-like symptoms, hematological effects, neuropsychiatric symptoms and various thyroid-related disorders, in patients with HCV infection [14]. Approximately 0.1 to 1% of patients who are treated with IFN are at risk of more complicated and life-threatening side effects, including thyroid, visual, auditory, renal and cardiac impairment as well as pulmonary interstitial fibrosis [13, 15]. The important immunomodulatory properties of IFN are due to its ability to induce autoimmune phenomena; however, that effect can result in patients developing autoimmune thyroid diseases, such as hypothyroidism or hyperthyroidism [16]. Up to 20% of patients suffering from autoimmune thyroiditis have been reported to receive IFN-based therapies [17]. Thyroid dysfunction caused by IFN treatment may also present various clinical pictures, such as destructive thyrotoxicosis, Graves’ thyrotoxicosis and hypothyroidism. These pathological conditions may be present in treated patients and be induced by the different immunological effects of IFN therapy on the thyroid gland [18]. IFN treatment may cause a defect in the function of intrathyroidal iodide organification, thus further reducing hormone synthesis [19].

The combination of IFN plus RBV for treating CHC is used in Taiwan [20]. It has been reported that treatment with IFN plus RBV for CHC leads to a sustained virologic response rate of 54 to 80% [21, 22]. The National Health Insurance Administration in Taiwan began to provide reimbursements for PGE-IFN/RBV treatment for CHC patients [22]. Since 2003, approximately 81,000 patients have been treated after this universal reimbursement was implemented [23]. Despite its success in treating HCV infection, the association between HCV infection treatment and thyroid disorders has made the treatment controversial. However, data on this subject are scarce in Taiwan. The aim of our study was to investigate the occurrence and risk factors of thyroid dysfunction in HCV-infected subjects in Taiwan.

## Methods

### Study population

The study methods had been published previously [22, 24]. Briefly, this study is based on data from the National Health Insurance Research (NHIR) database provided by the Taiwan National Health Research Institute. The National Health Insurance (NHI) program in Taiwan was implemented in March 1995. As of 2007, as many as 96% of the citizens in Taiwan had joined the NHI program [25]. The NHIR database contains every medical claim record (inpatient care and outpatient care) for the years 2001 to 2013, including sex, date of birth, date of visit, past history, and the International Classification of Disease, Ninth Revision, Clinical Modification (ICD-9-CM) diagnosis [26].

We followed all study subjects from the index date to the date of diagnosis of TD, death, or the end of this study, 31 December 2013, to compare the risk for developing TD between the case patients and control subjects. All study subjects who met the definition of a case patient before the index date were excluded. The index date was defined as the date when the patient was first diagnosed as a case patient.

For the protection of privacy, the patient and institute identifications were scrambled cryptographically in the case file to attain anonymity, ensuring that the individuals could not be identified individually.

### Definition

Cases were defined as patients with a confirmed CHC diagnosis (ICD-9-CM codes 07044 and 07054) that received PEG-IFN/RB treatment during admission or ambulatory care between 1 January 2001 and 31 December 2013. The control subjects were defined as patients who were diagnosed with CHC (ICD-9-CM codes 07044 and 07054) and did not receive treatment; controls were matched by the index date, age (± 3 years), and sex with cases at a ratio of 3:1.

Chronic hepatitis C infection was defined by ICD-9-CM codes 07044 and 07054. The thyroid dysfunctions included were hypothyroidism (ICD-9-CM codes 243 and 244), hyperthyroidism (ICD-9-CM code 242), and thyroiditis (ICD-9-CM codes 245 and 246).

Past history included the diseases probably associated with thyroid function, which were defined as follows before the index date, during one or more admissions or three or more ambulatory care visits: hyperlipidemia (ICD-9-CM code 272), diabetes mellitus (ICD-9-CM codes 250.0–250.91), hypertension (ICD-9-CM codes 401–405), chronic renal disease (ICD-9-CM codes 403, 404, 581–583, and 585–588), and liver cirrhosis (ICD-9-CM codes 571.5), and goiter (ICD-9-CM 240–241).

Hyperthyroidism, including subclinical hyperthyroidism, was defined as having a low TSH level (< 0.4 mIU/mL) [27, 28]. Hypothyroidism, including subclinical hypothyroidism, was defined as having a high TSH level (> 4.1 mIU/mL) [27, 28]. Thyroiditis was diagnosed when sudden hyperthyroidism occurred, then, after several days/weeks, hypothyroidism developed, and during the following weeks and months, the thyroid gland resumed its normal function or hypothyroidism remained [29].

Hyperlipidemia was defined as low-density lipoprotein cholesterol (LDL-C) ≥3.5 mmol/L (≥137 mg/dL) and/or total cholesterol (TC) ≥5 mmol/L (≥200 mg/dL) and/or triglycerides ≥3.9 mmol/L (≥150 mg/dL) and/or use of prescribed lipid-lowering medications [30].

### Ethical statement

We used the NHIR database in this study. According to the regulation of Ministry of Health and Welfare, Taiwan, all of the NHIR datasets are only available from the information Center, Ministry of Health and Welfare, Taiwan. This study was conducted according to the Declaration of Helsinki and was approved by the Institute Review Board of the Show Chwan Memorial Hospital, Taiwan (No. 1051201). Informed consent was waived because all data in this study were unidentifiable and encrypted. The rights and welfare of the study subjects were not affected.

### Statistical analyses

The annual incidence rate of TD cases was calculated by dividing the number of TD cases diagnosed by physicians by the follow-up person-years of the study patients. The annual incidence of TD cases was expressed as the number of cases with TD per 100,000 person-years. Comparisons between groups were performed using a chi-square test for categorical variables and a t-test for continuous variables.

The Kaplan-Meier method was used to evaluate the incidence rates from the first diagnosis of hepatitis C infection to the occurrence of TD. Cases were evaluated for the occurrence of a TD diagnosis at follow-ups through December 2013. Individuals who were event-free (no TD diagnosed) at the time of data censoring were assumed to have normal thyroid function. Differences in the occurrence of TD were assessed using a log-rank test [31].

We calculated the crude hazard ratios (HRs) in CHC patients for the occurrence of TD. The incidence and 95% confidence interval (CI) were also calculated. Multivariate Cox proportional hazard models were applied to analyze the association between PEG-IFN/RBV treatment and the incidence of TD after adjusting for age, sex, and comorbidities [31]. The data were analyzed using SPSS version 17.0 (SPSS Inc., Chicago, IL, USA). Two-tailed *p* values < 0.05 were considered significant.

### Results

#### Overall basic demographic and clinical characteristics of all cases with chronic hepatitis C infection

The characteristics of all patients with HCV infection are shown in Table 1. From 2001 through 2013, 13,203 CHC patients that met the case definition were enrolled in this study. Of these, 3810 (28.9%) were classified as cases, and 9393 (71.1%) were classified as controls. The male-to-female ratio was 0.96, and the median age was 46 years (range: 18–80 years). The highest number of cases was in the 51 years and older age group (41.3%), and the lowest was in the < 20 years age group (4.6%). The number of patients was different in different regions of Taiwan, with the highest number in the southern region (38.0%) and the lowest in the eastern region (6.5%). Of the study subjects, 417 (3.2%) were diagnosed with TD, and 12,786 (96.8%) were event-free. The number of patients with a past history of hypertension, hyperlipidemia, diabetes, liver cirrhosis, chronic kidney disease (CKD), and goiter were 6908 (52.3%), 4642 (35.2%), 4282 (32.4%), 2916 (22.1%), 2473 (18.7%), and 485 (3.7%), respectively. The mean follow-up period was 48 months (range: 6–140 months).

**Table 1:** Demographic characteristics of patients with CHC in Taiwan, 2001–2013

Variables	Total *N* = 13,203	CHC treated by PEG IFN/RB *N* = 3810	CHC nontreated *N* = 9393	*P*-value
	n (%)	n (%)	n (%)	
Age (years)				0.69
< 20	612 (4.6)	183 (4.8)	429 (4.6)	
21–30	2104 (15.9)	610 (16.0)	1494 (15.9)	
31–40	2607 (19.7)	723 (19.0)	1884 (20.1)	
41–50	2430 (18.4)	704 (18.5)	1726 (18.4)	
> 51	5450 (41.3)	1590 (41.7)	3860 (41.1)	
Sex				0.84
Female	6733 (51.0)	1980 (52.0)	4753 (50.6)	
Male	6470 (49.0)	1830 (48.0)	4640 (49.4)	
Region of residence				0.03
Northern	3549 (26.9)	948 (24.9)	2601 (27.7)	
Central	3772 (28.6)	1149 (30.2)	2623 (27.9)	
Southern	5019 (38.0)	1450 (38.1)	3569 (38)	
Eastern	863 (6.5)	263 (6.9)	600 (6.4)	
Diagnosed with TD				< 0.001
Yes	417 (3.2)	173 (4.5)	244 (2.6)	
No	12,786 (96.8)	3637 (95.5)	9149 (97.4)	
Past history				
Hypertension				< 0.001
Yes	6908 (52.3)	1759 (46.2)	5149 (54.8)	
No	6295 (47.7)	2051 (53.8)	4244 (45.2)	
Hyperlipidemia				0.901
Yes	4642 (35.2)	1383 (36.3)	3259 (34.7)	
No	8561 (64.8)	2427 (63.7)	6134 (65.3)	
Diabetes mellitus				< 0.001
Yes	4282 (32.4)	1164 (30.6)	3118 (33.2)	
No	8921 (67.6)	2646 (69.4)	6275 (66.8)	
Liver cirrhosis				0.003
Yes	2916 (22.1)	756 (19.8)	2160 (23.0)	
No	10,287 (77.9)	3054 (80.2)	7233 (77.0)	
CKD				< 0.001
Yes	2473 (18.7)	552 (14.5)	1921 (20.5)	
No	10,730 (81.3)	3258 (85.5)	7472 (79.5)	
Goiter				0.48
Yes	485 (3.7)	133 (3.5)	352 (3.7)	
No	12,718 (96.3)	3677 (96.5)	9041 (96.3)	

*CHC* chronic hepatitis C, *TD* thyroid dysfunction, *CKD* chronic kidney disease, *PEG-IFN/RBV* pegylated interferon/ribavirin

Table 1 Compared to the nontreated group, the treated group had a higher rate of TD and lower rates of hypertension, diabetes, liver cirrhosis, and CKD. The frequency of TD in patients treated with PEG-IFN/RB was significantly higher than that in nontreated patients (4.5% vs. 2.6%, *P* < 0.001). There were no significant differences between the treated and nontreated groups with respect to age, sex, past history of hyperlipidemia and goiter.

#### Distribution of thyroid dysfunction

Table 2 shows the distribution of TD types among the 417 patients with TD. Among them, 179 (42.9%) were diagnosed with hypothyroidism, 130 (31.2%) with hyperthyroidism, and 108 (25.9%) with thyroiditis. In the patients with TD, 173 (41.5%) had received PEG-IFN/RBV treatment, while 244 (58.5%) patients had not.

**Table 2:** Distribution of thyroid dysfunction types in patients with HCV infection in Taiwan, 2001–2013

Variables	Total number (%)	Nontreated group N (%)	Treated group^a^ N (%)
Hypothyroidism	179 (42.9)	104 (42.6)	75 (43.3)
Hyperthyroidism	130 (31.2)	74 (30.3)	56 (32.4)
Thyroiditis	108 (25.9)	66 (27.0)	42 (24.3)
Total	417 (100.0)	244 (100.0)	173 (100.0)

^a^ Treated group: patients treated with pegylated interferon and ribavirin

#### The correlation between PEG-IFN/RBV treatment and thyroid function

**Fig. 1:**
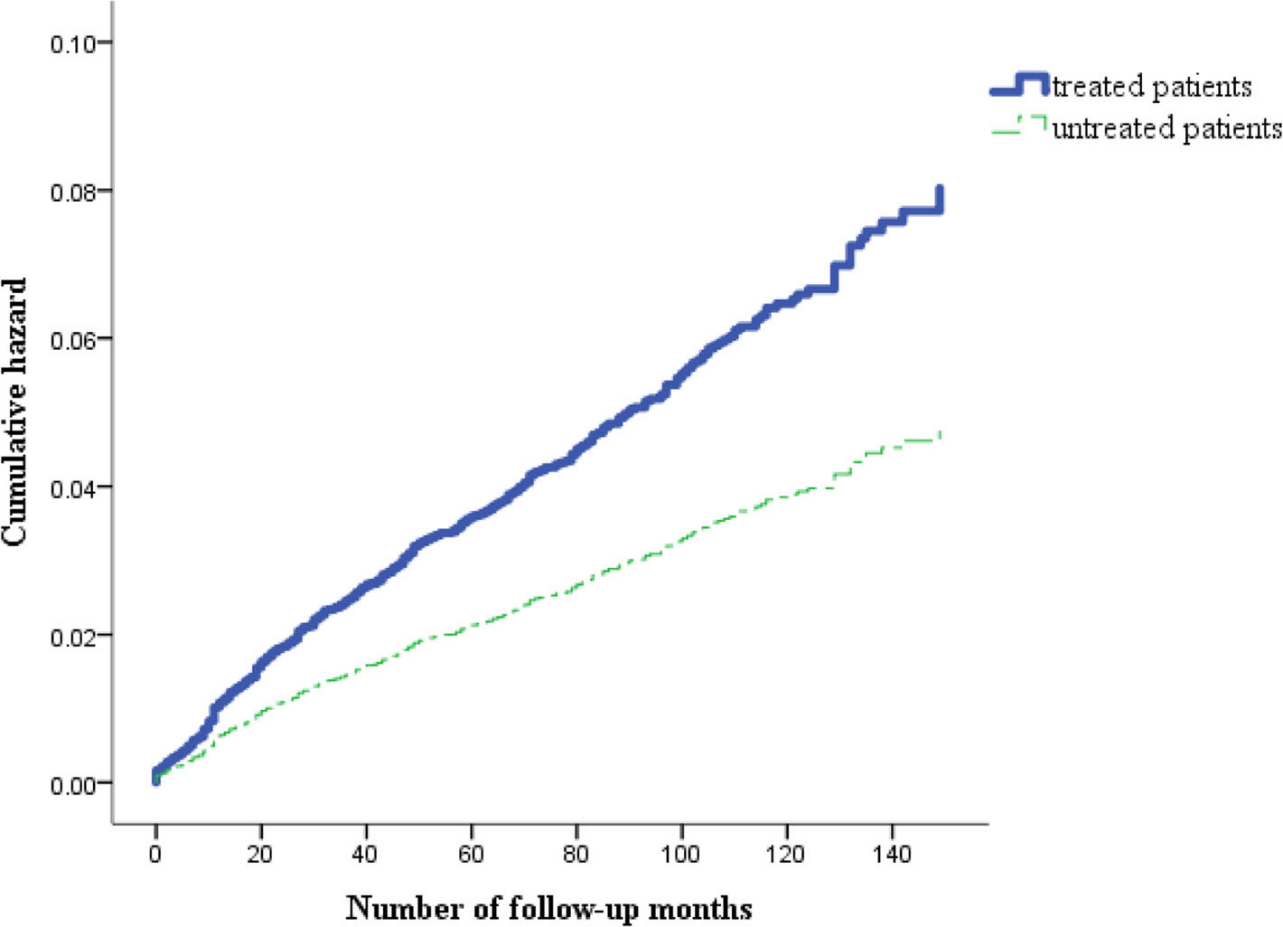
Cumulative hazard rate of thyroid dysfunction estimated by the Kaplan-Meier approach in patients treated with pegylated interferon/ribavirin and untreated patients in Taiwan, 2001–2013

During the study period, 173 (4.5%) CHC patients who had been treated with PEG-IFN/RBV were diagnosed with TD, corresponding to an incidence rate of 60.8 (range: 60.0–94.0) per 100,000 person-years. In the untreated group, 244 (2.6%) patients had TD, corresponding to an incidence rate of 36.9 (range: 37.7–53.3) per 100,000 person-years. Figure 1 shows the cumulative risk of the occurrence of TD among patients treated with PEG-IFN/RBV and untreated patients. For the occurrence of TD during the 13-year follow-up period, the patients treated with PEG-IFN/RBV were at a higher risk of suffering from TD (*P* < 0.0001), according to the Kaplan-Meier analysis.

#### Factors associated with thyroid dysfunction among patients with HCV infection

We performed a Cox regression analysis with forward selection procedures based on the likelihood ratio. As shown in Table 3, the factors of CHC treatment with PEG-IFN/RBV including female sex, hyperlipidemia and a history of goiter were associated with a higher risk of thyroid dysfunction in univariate analysis. Age, regional residence, and comorbidities, including hypertension, CKD, diabetes mellitus, and liver cirrhosis, were not associated with the occurrence of TD (all *P*-values > 0.05).

**Table 3:** Risk factors associated with TD among patients with hepatitis C infection by univariate and multivariate Cox regression analyses

	Univariate analysis		Multivariate analysis	
Variables	Hazard ratio (95% CI)	*P*-value	Hazard ratio (95% CI)	*P* value
Sex (F/M)	1.46 (1.19–1.78)	< 0.001	1.49 (1.23–1.75)	< 0.001
PEG-IFN/RBV treatment (yes/no)	1.70 (1.40–2.07)	< 0.001	1.68 (1.38–2.06)	< 0.001
Hyperlipidemia (yes/no)	141 (1.16–1.72)	< 0.001	1.38 (1.12–1.71)	< 0.001
Past history of goiter (yes/no)	6.61 (5.18–8.43)	< 0.001	6.40 (5.00–8.18)	< 0.001

*TD* thyroid dysfunction, *HR* hazard ratio, *CI* confidence interval, *M* males, *F* females, *PEG-IFN/RBV* pegylated interferon/ribavirin, *CKD* chronic kidney disease

The dependent variable was thyroid dysfunction (yes/no). The available independent variables were age, sex, PEG-IFN/RBV treatment, hyperlipidemia, regional residence, hypertension, CKD, diabetes mellitus, liver cirrhosis, past history of goiter

Multivariate analysis revealed that the strongest factors associated with a higher risk for the development of TD were female sex (adjusted hazard ratio (HR): 1.49; 95% confidence interval (CI): 1.23–1.75; *P* < 0.001), treatment with PEG-IFN/RBV (HR: 1.68; 95% CI: 1.38–2.06; *P* < 0.001), hyperlipidemia (HR: 1.38; 95% CI: 1.12–1.71; *P* < 0.001), and a history of goiter (HR: 6.40; 95% CI: 5.00–8.18; *P* < 0.001) (Table 3).

## Discussion

As our knowledge, this is the first study using NHIR database to assess the relationship between TD and PEG-IFN/RBV treatment in patients with CHC infection in Taiwan. The results presented in this study demonstrate that the frequency of TD in Taiwanese patients with CHC who were treated with PEG-IFN/RBV was higher than that in those who were not treated with PEG-IFN/RBV (4.5% vs. 2.6%; *P* < 0.001). We observed that the cumulative number of CHC patients with TD increased after the introduction of PEG-IFN/RBV treatment in 2003. Indeed, TD is a common endocrinopathy that is associated with IFN-based treatments of HCV infection [19, ﻿32﻿].

A previous study indicated that the most commonly recognized TDs among CHC patients treated with PEG-IFN/RBV were hyperthyroidism (45.5%), hypothyroidism (33.8%), thyroiditis (19.5%), and goiter (1.3%) [29]. Similar to the findings in the previous study [29], we found that the TDs identified among patients with HCV infection were hypothyroidism (42.9%), hypothyroidism (31.2%), and thyroiditis (25.9%).

Thyroid dysfunctions have been reported due to HCV itself and due to treatment with IFN-based drugs [13, 33]. HCV, which is mainly a hepatotropic virus, also causes various autoimmune diseases [34]. In fact, a significant proportion of CHC patients suffer from TD before IFN treatment [35]. There are some theories that have emphasized the important role that HCV plays in the sustained reaction of the immune system, e.g., the infection of lymphatic cells; the production of viral proteins, chromosomal aberrations, and cytokines; and cell molecule changes [36].

However, a considerable number of studies have found thyroid disorders in CHC patients treated with IFN-based drugs. The association of the IFN regimen with TD is quite important because evidence shows that as the IFN levels increase, the levels of autoimmune reactions, including the activation of both the innate and adaptive immune responses, also increase [13]. The recognized underlying molecular mechanisms include polymorphisms in the IFN signaling pathways, a feed-forward loop of IFN production, and a mutually positive regulatory feedback loop between IFN and estrogen receptors [37]. The mechanism for the development of thyroid autoimmunity after treatment with IFN/RBV may be the activation of the Th1 pathway during the immune response, which then induces cell-mediated cytotoxicity [34]. Additionally, thyroiditis induced by interferon is a substantial clinical problem for patients who are treated with IFN, with complications of thyrotoxicosis occurring in patients with severe or life-threatening complications [17, 38]. In clinics, reducing the dose or discontinuing IFN therapy in patients who develop TD has been used to alter the therapeutic response of IFN treatment [19]. Several studies have demonstrated that TD is mediated by indirect immunological reactions rather than directly by HCV invasion [13, 17, 38, 39], which may be due to a malfunction of self-tolerance and the consequent triggering of the autoimmune response. Similar to the results of previous studies [40–42], our study found that HCV-infected patients treated with PEG-IFN/RIBV were at a higher risk of developing TD than were patients who did not receive treatment.

A previous study showed that the frequencies of newly developed TD during IFN treatment (monotherapy) and combined therapy were 2.7 and 12.8%, respectively [13, 33, 43]. Compared to those reported previously, our study showed a lower incidence (4.5%) of IFN-induced TD in patients with CHC. The inconsistency may be due to the definition of TD, study population ethnicity, or regional differences in iodine status among the study subjects [44]. For example, if a patient developed TD several years after the completion of treatment, should this case be classified as related to the treatment with interferon or not? In addition, the clinical characteristics of TD are often subclinical and masked by the effects of IFN therapy [13].

Although it has been found that some patients with CHC may have TD before treatment [7, 45], it is believed that HCV itself may predispose patients to the development of TD. The most common hypothesis for the endocrine side effects of these IFN-α-based regimens, outside of the liver, is the production of autoantibodies that induce the development of TD [7, 46–48]. However, we could not suggest this theory because our study did not collect data regarding sustained virologic response (SVR) among the studied patients.

A previous study showed that thyroid disorders are more common in females and that female sex is a predictive risk factor for TD during PEG-IFN/RBV treatment [32]. Similar to the previous study, it was found in this study that females had a higher risk for developing TD than males did.

Our study found that patients with hyperlipidemia were at a higher risk for developing thyroid disorders. It has been reported that thyroid hormones are the main regulators of total cholesterol (TC) and lipoprotein-cholesterol metabolism [49]. This biological relationship may explain the impact of hypothyroidism, which is often linked to excess TC and low-density lipoprotein cholesterol (LDL-C) [50]. It should be noted that endocrine disorders, including hypothyroidism and type 2 diabetes mellitus, may induce or exacerbate existing dyslipidemia and impede the achievement of optimal results through hypolipidemic drugs [51]. Therefore, CHC patients with dyslipidemia should be regularly checked for thyroid dysfunction, and both cholesterol and its subfractions should be monitored in CHC patients undergoing PEG-IFN/RBV treatment with thyroid failure [49, 51].

Lipid metabolism is an important liver function. Patients with HCV infection have lower lipid profiles [52]. Several studies have reported that HCV infection is associated with lower lipid profiles and progression to dyslipidemia, liver steatosis or advanced fibrosis [52, 53]. A study in Taiwan showed that the clearance of HCV RNA is the main determinant for the increase in lipids after PEG-IFN/RBV treatment [54]. In addition, thyroid hormones are known to be the main regulators of total cholesterol (TC) and lipoprotein cholesterol metabolism [55]. Therefore, the impact of hypothyroidism is often linked to excess TC and low-density lipoprotein cholesterol (LDL-C) [56]. The roles of HCV and thyroid hormones on lipid metabolism needs to be further investigated in the future.

Our study found that patients with a history of goiter were at higher risk for developing thyroid dysfunction. Patients with goiter, an enlargement of the thyroid gland, may have symptoms of normal thyroid function (nontoxic goiter), decreased thyroid function (hypothyroid goiter), or increased thyroid function (hyperthyroid goiter) [57]. The reasons for the relationship between a history of goiter and TD among CHC patients treated with PEG-IFN/RB remain unclear. A previous study indicated that the most common side effect of PEG-IFN/RB treatment is the production of autoantibodies and the development of TD [45]. Although we did not have data on autoantibodies in this study, a history of goiter may be a surrogate factor for previous thyroid disorders.

A strength of this study was the large study population. However, our study had several limitations. First, observational studies, such as this one, should be interpreted with caution. Second, the data were collected primarily according to financial claims, which could potentially affect the quality of the data to some degree. Socioeconomic factors (e.g., education, smoking) and molecular data (e.g., genotypes) were not available. Third, diagnoses of comorbidities (e.g., CKD) listed in this study were not confirmed by laboratory or imaging data. Despite these limitations, the data from this observational study on patients treated with PEG-IFN/BV could provide useful information.

In summary, we present the epidemiologic characteristics of TD and its risk factors among patients with CHC in Taiwan. The results demonstrate that the prevalence of TD was 4.5%. TD was significantly associated with PEG-IFN/RBV treatment and hyperlipidemia in patients with CHC.

## Conclusions

Our study confirmed the association of thyroid dysfunction and PEG-IFN/RBV treatment for chronic HCV infection. Our results suggest that clinicians should monitor thyroid function in patients with chronic HCV infection who are being treated with PEG-IFN/RBV therapy, especially those who are female and/or have a history of hyperlipidemia or goiter.

## Abbreviations

CHC: Chronic hepatitis C
CKD: Chronic kidney disease
HCV: Hepatitis C virus
NHI: National Health Insurance
NHIR: National Health Insurance Research
PEG-IFN/RBV: Pegylated interferon/ribavirin
RBV: Ribavirin
TD: Thyroid dysfunction
